# Neurotransmitter-mediated gut-brain axis: a bibliometric analysis of research trends and knowledge structure

**DOI:** 10.3389/fmicb.2026.1771169

**Published:** 2026-04-13

**Authors:** Ziyi Guo, Yanchen Feng, Zhuoyu Ren, Wenbin Wang, Ruoyao Huang, Jie Zhao

**Affiliations:** 1 National Engineering Laboratory for Internet Medical Systems and Applications, The First Affiliated Hospital of Zhengzhou University, Zhengzhou, Henan, China; 2 Department of Rehabilitation Medicine, The First Affiliated Hospital of Zhengzhou University, Zhengzhou, Henan, China; 3 School of Basic Medical Sciences, Heilongjiang University of Chinese Medicine, Harbin, China; 4 Department of Anesthesiology, Pain and Perioperative Medicine, The First Affiliated Hospital of Zhengzhou University, Zhengzhou, China; 5 Department of Pharmacy, The First Affiliated Hospital of Zhengzhou University, Zhengzhou, Henan, China; 6 Hospital Office, The First Affiliated Hospital of Zhengzhou University, Zhengzhou, Henan, China

**Keywords:** gut–brain axis, gut microbiota, neurotransmitter, bibliometric, neuroimmune, depression

## Abstract

**Introduction:**

The gut–brain axis constitutes a bidirectional network linking the gastrointestinal tract and central nervous system through neural, endocrine, metabolic, and immune pathways. Neurotransmitters play a central role in mediating this crosstalk, serving as intermediates through which the gut microbiota influences brain function. Although important mechanistic advances have been made, research on the neurotransmitter-mediated gut–brain axis remains fragmented across disciplines. This study aimed to provide a comprehensive bibliometric overview of this field.

**Methods:**

We conducted a bibliometric analysis of 788 publications retrieved from Web of Science, Scopus, and PubMed between 2005 and 2025. Using VOSviewer, CiteSpace, and Pajek, we analyzed publication trends, geographic distribution, institutional and author contributions, journal co-citations, and keyword evolution to characterize the knowledge structure and emerging themes of the field.

**Results:**

The results revealed three developmental phases: an exploratory phase (2005–2016) with limited output, a developmental phase (2017–2019) with moderate growth, and a rapid expansion phase (2020–2025) marked by exponential increases in publications driven by advances in microbiome and neurotransmitter research. China led in publication volume, while the United States and Ireland served as major hubs of collaboration. University College Cork showed the highest citation impact, with 10,935 citations from 28 publications (average citations per document = 390.54). John F. Cryan, Timothy G. Dinan, and Gerard Clarke were among the leading contributors, with Cryan ranking first in both publication output and citation count. Keyword and thematic analyses identified gut microbiota, serotonin, short-chain fatty acids, depression, and inflammatory bowel disease as core topics, reflecting a shift from mechanistic studies to disease-specific and neurotransmitter-targeted research. Highly cited studies focused on microbial regulation of neurotransmitters, neuroimmune signaling, and their implications for neurodevelopmental and neurodegenerative disorders.

**Discussion:**

This study provides the first comprehensive bibliometric overview of neurotransmitter-mediated gut–brain axis research, offering a macroscopic perspective on its evolution, core knowledge base, and emerging frontiers. Future research should integrate multidisciplinary approaches, apply omics technologies, and develop precision interventions targeting neurotransmitter pathways while considering individual microbial profiles, in order to translate mechanistic insights into therapeutic strategies for neuropsychiatric disorders.

## 1 Introduction

The gut-brain axis is a complex bidirectional communication network that connects the gastrointestinal tract and the central nervous system through neural, endocrine, metabolic, and immune pathways (He et al., 2024). This system enables the central nervous system to integrate interoceptive signals from the gut and coordinate adaptive responses, thereby playing a critical role in maintaining host homeostasis (Osadchiy et al., 2019). Traditionally, research on gut–brain interactions focused on visceral sensation, autonomic regulation, and digestive function (Wang et al., 2023). However, advances in microbiome science over the past research have reshaped this framework, revealing that the gut microbiota acts as a key regulator within the gut-brain axis (Schneider et al., 2024). Beyond its classical metabolic functions, the microbial ecosystem produces diverse bioactive molecules and modulates host pathways that influence neuronal excitability, synaptic plasticity, stress responsivity, and emotional behavior (Vicentini et al., 2021; Chen et al., 2021; Muller et al., 2020; Liu et al., 2023; Agirman et al., 2021). These discoveries have made the gut-brain axis a rapidly expanding interdisciplinary research frontier, covering neuroscience, microbiology, psychiatry, nutrition, immunology, and systems biology.

Among the multiple pathways mediating gut–brain communication, neurotransmitter-related mechanisms have emerged as particularly direct and influential routes. Gut microbes can synthesize neuroactive compounds such as *γ*-aminobutyric acid and dopamine, and influence host neurotransmitter systems, for example, by modulating the tryptophan–serotonin pathway (Valles-Colomer et al., 2019). Microbial metabolites, including short-chain fatty acids, indole derivatives, and bile acid metabolites, regulate enteroendocrine signaling, enteric neuronal activity, and microglial function, thereby affecting central neurotransmission (Silva et al., 2020; Ahmed et al., 2022). These neurochemical signals reach the brain via vagal afferents, circulatory transport, and immune-mediated pathways, ultimately modulating synaptic transmission, neuroinflammation, and neural circuit function. Because neurotransmitters operate at the core of neuronal communication, their involvement provides a mechanistic bridge linking gut microbiota with brain function (Dohnalová et al., 2022). Therefore, neurotransmitter-mediated pathways represent a central focus in contemporary gut-brain axis research.

Despite growing recognition of the importance of neurotransmitter pathways, the literature has become conceptually rich but increasingly fragmented. Studies vary widely in disciplinary background, methodological approaches, neurotransmitter systems examined, and disease contexts. While several narrative reviews have summarized mechanistic insights, no comprehensive bibliometric analysis has systematically mapped the knowledge structure, research hotspots, collaborative networks, or temporal evolution of this domain. Without such an integrative overview, it remains challenging for researchers to identify converging themes and emerging frontiers.

To address this gap, the present study conducts a systematic bibliometric analysis of research at the intersection of the gut–brain axis and neurotransmitter-related mechanisms. Using quantitative mapping and visualization techniques, we aim to identify global trends, influential authors and institutions, thematic clusters, and emerging research directions. By providing a comprehensive overview of the intellectual landscape, this study offers an integrated framework to guide future mechanistic and translational investigations in neurotransmitter-focused gut–brain axis research.

## 2 Methods

### 2.1 Literature sources and search strategy

A systematic search was conducted in Web of Science Core Collection (WoSCC), Scopus, and PubMed. The search date was 1 November 2025, and the time span was limited to 1 January 2005 to 1 November 2025 (therefore, 2025 represents an incomplete year). All records were exported on 1 November 2025. All citation counts and derived citation-based indicators reported in this study reflect the values available in the databases on the extraction date and may change with subsequent indexing and citation accrual. Only English-language articles and reviews were included. Bibliographic fields rather than full text were searched: TS (Topic) in WoSCC (Title/Abstract/Author Keywords/Keywords Plus), TITLE-ABS-KEY in Scopus, and Title/Abstract in PubMed. The exact database-specific search strings (including field tags and filters) are provided in Supplementary Table S1.

### 2.2 Data integration, inclusion, and exclusion criteria

Data retrieved from the three databases were consolidated in Microsoft Excel 2021. For PubMed records, missing metadata (e.g., author keywords, abstracts, and institutional affiliations) were manually supplemented when feasible to improve completeness. Duplicate records across databases (including overlapping indexing between WoSCC and Scopus) were removed using a hierarchical strategy: (i) matching unique identifiers (DOI as the primary key; PMID and/or database accession numbers when available); (ii) exact matching of normalized titles (case-insensitive, with punctuation and whitespace standardized); and (iii) fuzzy title matching combined with first author and publication year for records lacking identifiers. All candidate duplicates identified by fuzzy matching were manually verified. When duplicates were confirmed, preferentially the WoSCC record when cited-reference fields were required for co-citation analyses—to avoid double counting. Publications of non-peer-reviewed or otherwise non-research types—including meeting abstracts, editorials, letters, conference proceedings, book chapters, and retracted publications—were excluded. Two authors independently screened titles and abstracts, with full-text review conducted as necessary to ascertain eligibility. Discrepancies were resolved through discussion with a third author.

### 2.3 Data processing and analysis

Bibliometric analyses were performed using quantitative and statistical methods to characterize the current research landscape, identify prevailing research hotspots, and anticipate emerging trends. Microsoft Excel 2021 was used to visualize annual publication trends, while R Studio facilitated mapping of research output and collaboration patterns across countries and regions. VOSviewer was employed to construct co-occurrence networks, and CiteSpace was used for in-depth analysis of keywords and other bibliometric indicators. Pajek was utilized for layout optimization of large-scale networks exported from VOSviewer, without altering underlying data metrics, solely to enhance visualization clarity. To quantify the potential impact of partial-year coverage in 2025, we conducted sensitivity analyses by refitting the publication growth model using data up to 2024 (excluding 2025) and comparing model parameters and goodness-of-fit.

## 3 Results

**Figure 1:**
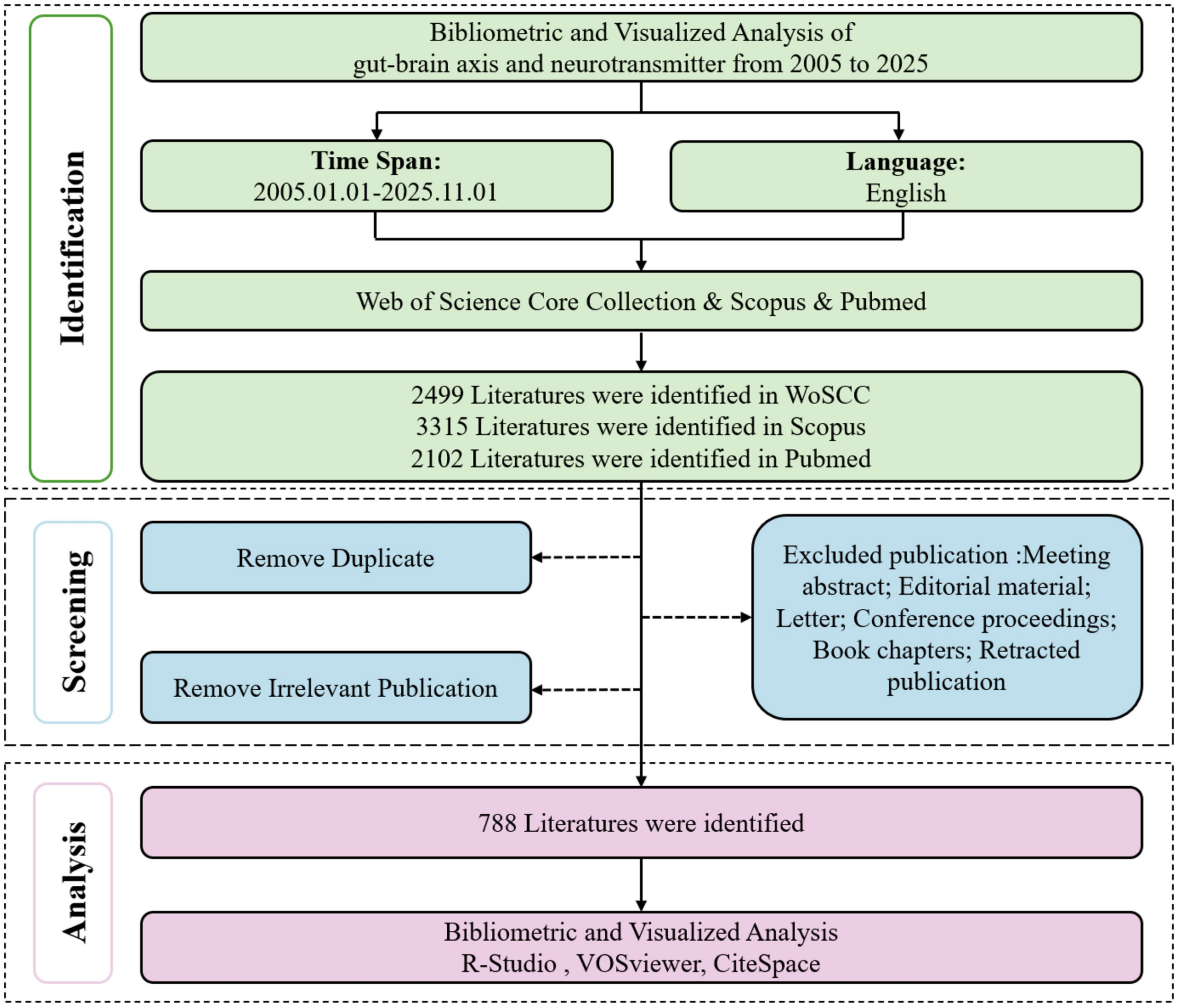
Flowchart of data collection for included studies.

A total of 7,916 records were initially identified through searches of Web of Science, Scopus, and PubMed, covering the period from 2005 to 2025. Among these, 2,499 records were retrieved from Web of Science, 3,315 from Scopus, and 2,102 from PubMed. After removing duplicates and screening titles and abstracts to exclude irrelevant publications, 788 articles were retained for subsequent analysis (Figure 1). The final dataset comprised 4,743 authors affiliated with 1,070 institutions across 47 countries, with an average of 58.89 citations per article.

### 3.1 Publication trends

**Figure 2:**
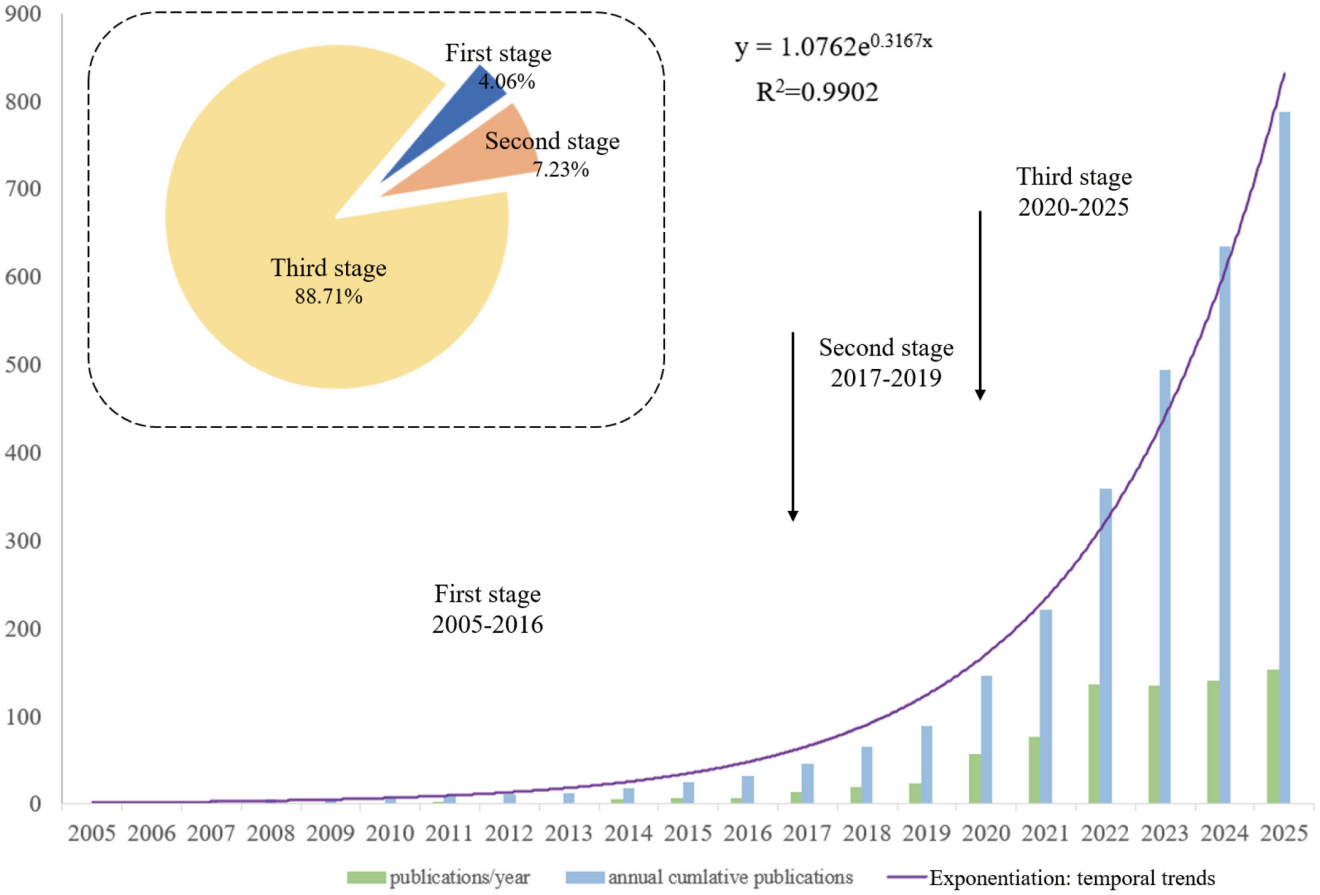
Temporal distribution of publications from 2005 to 2025 (data extracted up to 1 November 2025; 2025 represents a partial year).

The annual number of publications was calculated according to the year of publication. As shown in Figure 2, the development of annual and cumulative publications in this field from 2005 to 2025 can be divided into three stages. Initial Stage (2005–2016): During this period, the annual number of publications remained below 10, and cumulative growth was slow. Development Stage (2017–2019): Annual publications experienced a noticeable increase, rising from 7 articles in 2016 to 14 in 2017, and continued to grow rapidly to 24 articles in 2019. Rapid Growth Stage (2020–2025): After 2020, the annual number of publications exhibited exponential growth. It is important to note that the exponential growth model presented in this study is strictly descriptive and reflects historical publication trends, rather than making predictions about future research output. The cumulative publication trend was fitted with a linear regression model, yielding 𝑦=1.0762𝑒^0.3167𝑥^, 𝑅^2^ = 0.9902. where *x* denotes the number of years since 2005. Because 2025 represents a partial year (search cut-off: 1 November 2025), we conducted a sensitivity check by refitting the same exponential model after excluding 2025. As a sensitivity check, refitting the same model after additionally including the partial year 2025 (data extracted up to 1 November 2025) produced $y=1.1708e^{0.3241x}$ ( $R^{2}=0.9858$ ), indicating minimal changes in parameter estimates and an unchanged three-phase pattern. Over the past decade, research in this field has undergone substantial expansion. The exponential fit is intended as a descriptive summary of historical growth; formal regression assumption tests (e.g., residual normality/homoscedasticity) were not used to support inferential claims, and the model is not interpreted as a deterministic forecast, especially given the incomplete year 2025. In addition, alternative growth models (e.g., logistic/saturation or Gompertz-type curves, or segmented/joinpoint regression) may better capture long-term maturation and potential plateauing of the field over longer time horizons. Future updates with complete annual data could compare model fits rather than relying on a single functional form.

### 3.2 Analysis of key countries and regions

**Figure 3:**
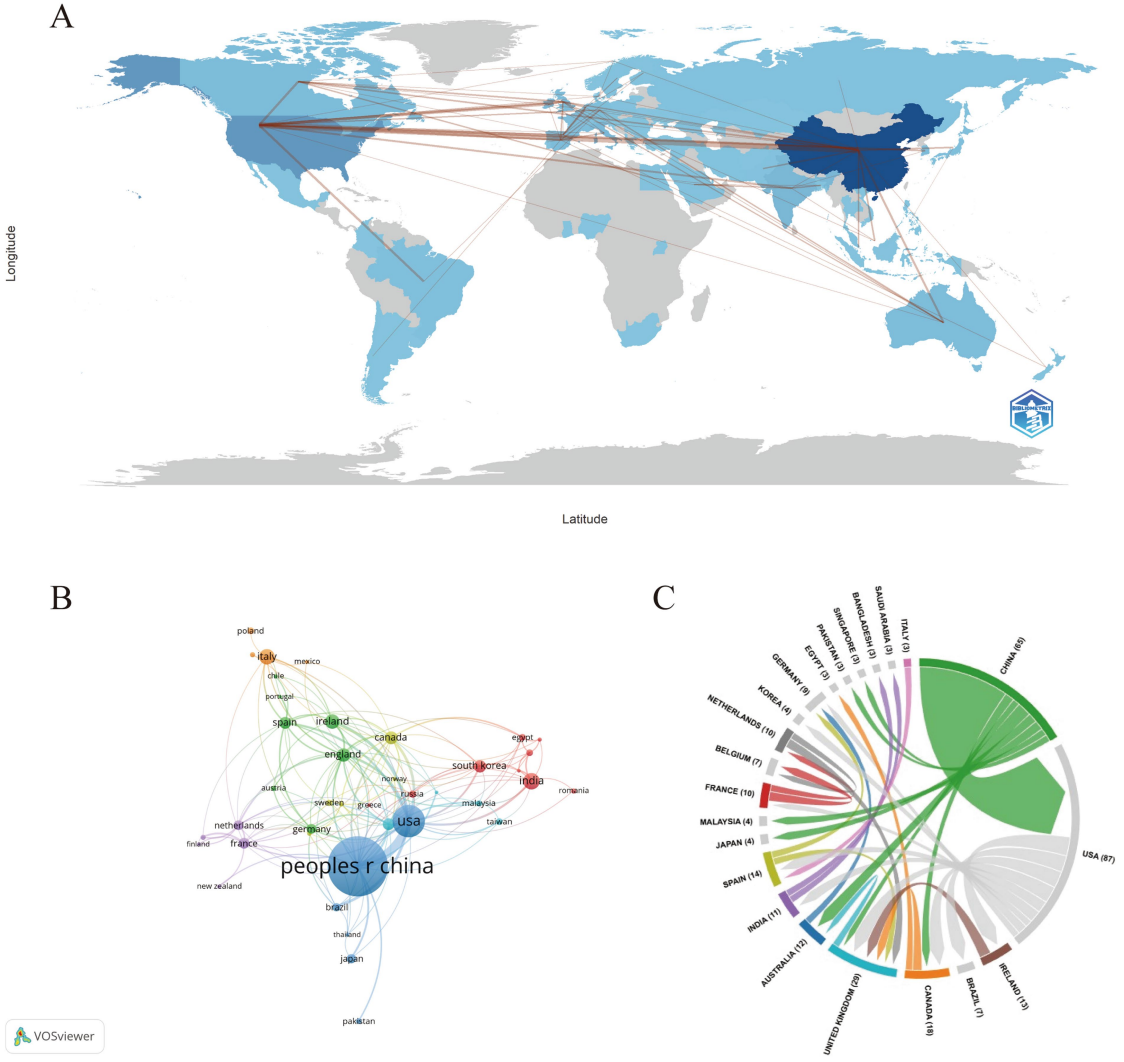
**(A)** World map showing the geographic distribution of publications. Darker shading indicates higher publication output, while gray indicates no/very low output in the dataset. Lines represent international co-authorship links, and line thickness indicates collaboration strength. **(B)** Country co-authorship network. Each node represents a country/region; node size is proportional to the number of publications, link thickness represents collaboration strength (total link strength), and node color indicates clusters (communities) identified by VOSviewer. **(C)** Chord diagram of bilateral collaborations among major contributing countries/regions. Arc length reflects each country’s total collaboration volume, and ribbon width reflects the strength of bilateral collaboration; ribbon colors correspond to the originating country/region.

Figure 3A shows the global geographic distribution of publications in this field. The international collaboration network (Figure 3B) summarizes country/region-level productivity and collaboration structure. In this network, nodes represent countries/regions, node size is proportional to publication counts, edge thickness represents collaboration strength (total link strength, TLS), and node color reflects average citations per country/region. In terms of research output, China contributed the largest number of publications (*n* = 375), followed by the United States (*n* = 133) and India (*n* = 43); Italy (*n* = 39) and Ireland (*n* = 35) ranked fourth and fifth, respectively (Table 1). Regarding collaboration connectivity, the United States showed the highest TLS (103), followed by China (87) and England (45), with Canada (33) and Spain (28) ranking next. Citation impact was assessed using both total citations and a normalized indicator, citations per publication (CPP = citations/documents), to reduce reliance on raw totals. Ireland ranked highest in total citations (14,449), followed by China (13,580) and the United States (9,315). When normalized by publication volume, Ireland also showed the highest CPP (412.83), whereas China showed a lower CPP (36.21) despite the largest publication output (Table 1), indicating different national profiles of “high citation yield” versus “high production volume.” Figure 3C further presents bilateral collaboration patterns, with the China–United States link being the strongest, reflecting intensive co-authorship between these two countries/regions within the dataset.

**Table 1:** Top 10 countries/regions by publication volume.

Rank	Country	Documents	Citations	TLS	CPP
1	China	375	13,580	87	36.21
2	USA	133	9,315	103	70.04
3	India	43	905	21	21.05
4	Italy	39	2,477	17	63.51
5	Ireland	35	14,449	26	412.83
6	England	31	2,148	45	69.29
7	Canada	29	7,556	33	260.55
8	South Korea	28	445	15	15.89
9	Spain	26	1,165	28	44.81
10	Australia	26	1,258	27	48.38

CPP, citations per publication (citations/documents); TLS, total link strength.

### 3.3 Core authors

**Figure 4:**
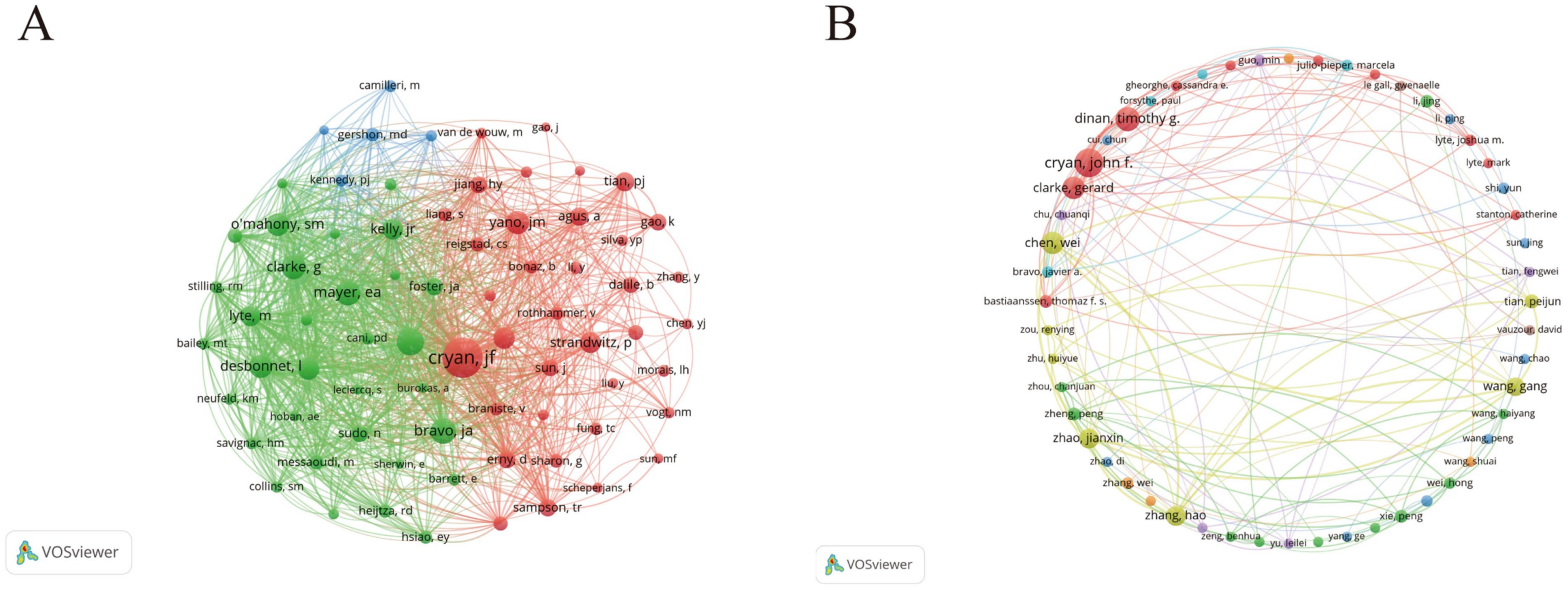
Author co-authorship networks in neurotransmitter-related gut–brain axis research. **(A)** Full author co-authorship network. Each node represents an author; node size is proportional to publication count, and links indicate co-authorship, with thicker links representing stronger collaboration (higher co-authorship link strength). **(B)** Co-authorship network among high-output authors (thresholded subset for clarity). Visual encodings are the same as in panel **(A)**: node size = publication count, link thickness = collaboration strength, and colors = clusters.

Analysis of authorship in the literature highlights the key researchers and leading contributors in this field. As shown in Figure 4A, nodes represent individual authors, with node size corresponding to the number of publications. Among them, John F. Cryan from University College Cork (UCC), Ireland, has published the highest number of articles, with a total of 28 publications, followed by Timothy G. Dinan and Gerard Clarke, who are affiliated with the same institution and rank second and third, respectively. In terms of publication output, Chinese researchers Chen Wei and Zhao Jianxin rank fourth and fifth. Notably, seven of the top 10 most prolific authors are from China, indicating a high level of research activity among Chinese scholars in this domain. Citation frequency, an important indicator of academic influence, reflects the publication rankings. The three authors with the highest citation counts are John F. Cryan, Timothy G. Dinan, and Gerard Clarke. These authors are identified as the most prominent contributors based on both publication output and citation impact. Paul Forsythe from McMaster University, Canada, ranks fourth, and Javier A. Bravo from Pontificia Universidad Católica de Valparaíso, Chile, ranks fifth in terms of citations. Figure 4B further visualizes these high-output and highly cited authors within the co-authorship network. In this visualization, node size reflects publication output and the structural prominence (connectivity) of authors within the co-authorship network, while link thickness represents collaboration strength.

### 3.4 High-contributing institutions

**Figure 5:**
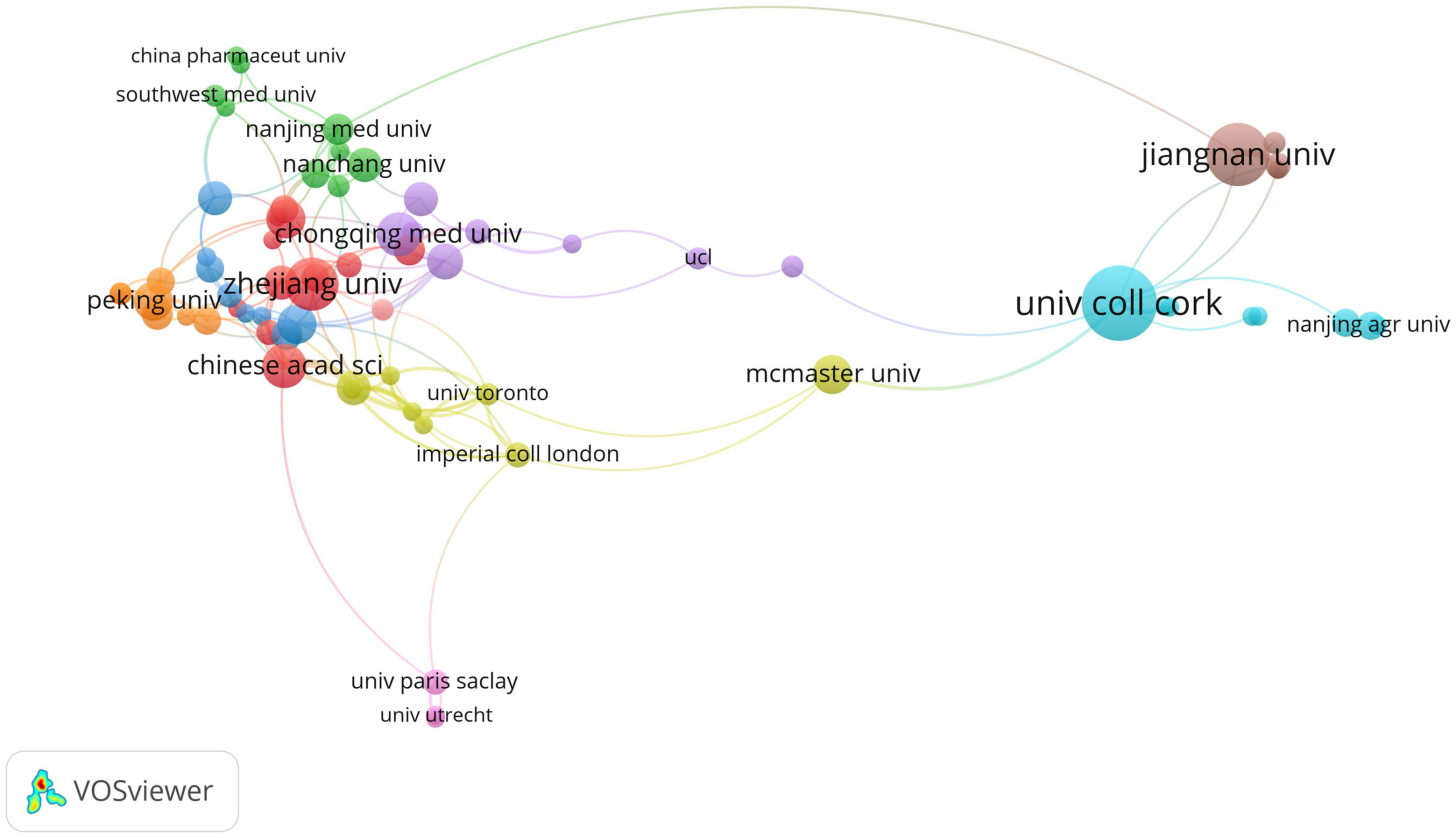
Institutional co-authorship network in neurotransmitter-related gut–brain axis research. Each node represents an institution; node size is proportional to the number of publications. Links indicate co-authorship collaboration between institutions, with thicker links representing stronger collaboration strength (higher co-authorship link strength).

Among the top 10 contributing institutions (Table 2), most are located in China, with additional contributions from Ireland and Canada. University College Cork (UCC), Ireland, ranks first in citation impact, with 28 publications and a total of 10,935 citations, yielding an average of 390.54 citations per publication. This high average citation rate highlights the strong citation impact of UCC relative to its publication volume. In comparison, Chinese institutions dominate in terms of publication volume. Jiangnan University and Zhejiang University rank second and third, respectively, with 22 publications and 1,521 citations and 17 publications and 649 citations. Chinese Academy of Sciences and Chongqing Medical University hold the fourth and fifth positions. Notably, institutions like McMaster University (Canada) and Peking University (China) exhibit high average citation counts, with McMaster University leading at 592.09 citations per publication. As shown in Figure 5, this distribution further emphasizes that research in this field is predominantly led by Chinese institutions, while UCC stands out for its high citation influence, reflecting a strong scholarly impact relative to its publication output.

**Table 2:** Top 10 institutions of high contribution.

Organization	Country	Documents	Citations	Average citations
University College Cork	Ireland	28	10,935	390.54
Jiangnan University	China	22	1,521	69.14
Zhejiang University	China	17	649	38.18
Chinese Academy of Sciences	China	13	1,633	125.62
Chongqing Medical University	China	13	1,052	80.92
McMaster University	Canada	11	6,513	592.09
Peking University	China	11	261	23.73
Shanghai Jiao Tong University	China	11	171	15.55
Southern Medical University	China	11	490	44.55
Hunan University of Chinese Medicine	China	10	106	10.60

### 3.5 High-contributing journals

**Figure 6:**
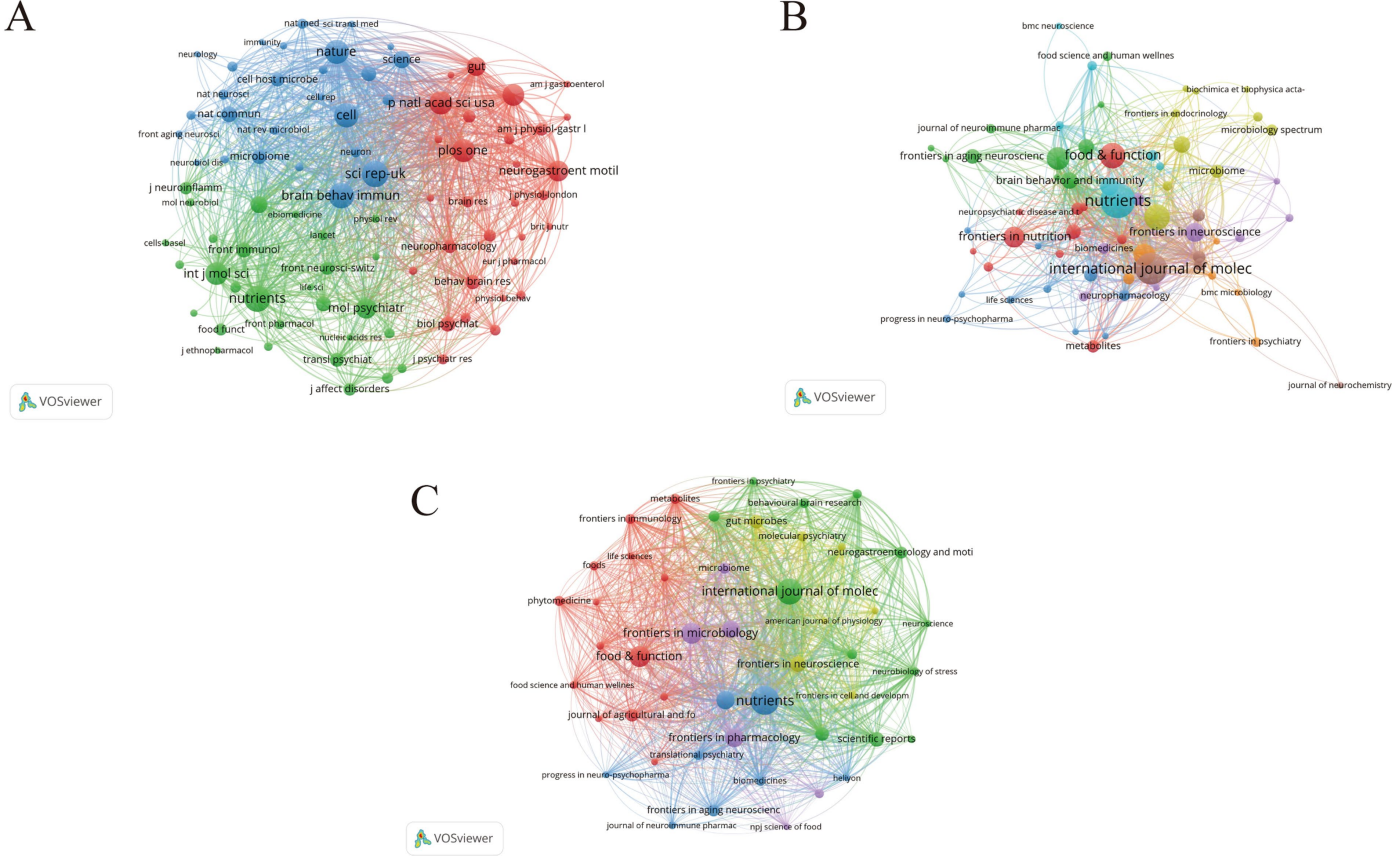
Journal-level knowledge structure and relatedness networks. **(A)** Journal co-citation network. Each node represents a journal; node size is proportional to co-citation frequency, and links represent co-citation relationships, with thicker links indicating stronger co-citation strength. **(B)** Network of leading journals. Each node represents a journal; node size reflects journal output/citation impact as configured in VOSviewer, and links indicate journal relatedness within the network. Colors denote clusters. **(C)** Document coupling network of journals, emphasizing similarity based on shared references. Each node represents a journal; links indicate coupling strength (shared references), with thicker links indicating stronger coupling. Node colors denote clusters, highlighting groups of journals with similar reference bases.

Analysis of journal co-citations reveals the long-established core knowledge structure within this field, as illustrated in Figure 6A. The clustered journal groups represent the foundational literature that is frequently cited collectively by the research community. The top 10 cited journals are listed in Table 3, among which six are classified as Q1 journals, indicating that they constitute the principal sources of high-impact publications in this domain. Nutrients leads in publication volume, with 31 articles, and also ranks highest in citations, with 2,403 citations. This highlights the journal’s sustained influence within the field, a pattern further confirmed by the visualization in Figure 6B. Document coupling analysis, which emphasizes recently published literature, is shown in Figure 6C. The journals with the largest nodes are distributed across multiple disciplines, including psychology, neuroscience, immunology, microbiology, and nutrition, indicating that the current research frontier in this field is characterized by a highly interdisciplinary and integrative knowledge network.

**Table 3:** Top 10 citing and cited sources.

Category	Journal	*N*	Total citations	Average citations	JCR 2025	Discipline
Citing sources	Nutrients	31	2,403	77.52	Q1	Nutrition and dietetics
	Brain behavior and immunity	11	2,179	198.09	Q1	Immunology
	Gastroenterology	3	1979	659.67	Q1	Gastroenterology and hepatology
	Molecular psychiatry	7	1835	262.14	Q1	Biochemistry and molecular biology
	Neurogastroenterology and motility	9	1,614	179.33	Q2	Clinical neurology
	Behavioral brain research	7	1,473	210.43	Q2	Behavioral sciences
	Neurobiology of stress	5	1,441	288.20	Q2	Neurosciences
	International journal of molecular sciences	27	1,415	52.40	Q1	Biochemistry and molecular biology
	Neuroscience	4	711	177.75	Q2	Neurosciences
	Neuropharmacology	6	700	116.67	Q1	Neurosciences
Cited sources	Nutrients	31	2,403	77.52	Q1	Nutrition and dietetics
	International journal of molecular sciences	27	1,415	52.41	Q1	Biochemistry and molecular biology
	Food and function	20	352	29.20	Q1	Biochemistry and molecular biology
	Frontiers in microbiology	20	584	17.60	Q1	Microbiology
	Frontiers in pharmacology	17	338	19.88	Q1	Pharmacology and pharmacy
	Frontiers in cellular and infection microbiology	16	594	37.13	Q1	Immunology
	Frontiers in nutrition	15	201	13.40	Q1	Nutrition and dietetics
	Frontiers in neuroscience	13	500	38.46	Q2	Neurosciences
	Scientific reports	12	645	53.75	Q1	Multidisciplinary sciences

### 3.6 Highly cited references

Highly cited references typically form the foundational knowledge base of a research field. An analysis of the 10 most frequently cited publications in this domain is presented in Table 4. Geographically, these seminal works are concentrated, with five originating from research institutions in Ireland, underscoring the country’s foundational contributions to the field. In terms of document type, the distribution is balanced, comprising six original research articles and four review papers, reflecting a combination of empirical exploration and systematic theoretical integration. Further content analysis indicates that these highly cited publications collectively construct a comprehensive scientific framework linking the microbiota–gut–brain axis with neurotransmitter systems across multiple levels, including molecular mechanisms, neural pathways, and overall behavioral outcomes.

**Table 4:** Top 10 most cited references.

Cited reference	Citations	Country	Year	Citations per year	type	Descriptions
Bravo et al. (2011)	186	Ireland	2011	13.29	Article	Beneficial bacteria in the gut directly regulate the expression of key neurotransmitter receptors in the brain via the vagus nerve.
Yano et al. (2015)	174	USA	2015	17.40	Article	Gut microbiota plays a critical role in regulating the production of the majority of the body’s 5-HT.
Cryan et al. (2019)	152	Ireland	2019	21.71	Review	Gut microbiota bidirectionally communicates with the brain via multiple pathways, making it a key therapeutic target for future neuropsychiatric disorders.
Cryan and Dinan (2012)	125	Ireland	2012	9.62	Review	The microbiota-gut-brain axis regulates anxiety, emotion, cognition and pain perception
Agus et al. (2018)	114	France	2018	16.29	Review	Tryptophan metabolism is the core link in the dialog between intestinal microbiota and the host
Clarke et al. (2013)	113	Ireland	2013	9.42	Article	Early-life gut microbiota deficiency critically shapes central nervous system 5-HT signaling
O'mahony et al. (2015)	100	Ireland	2015	10.00	Review	Gut microbiota regulates gut–brain axis function mainly by modulating serotonergic balance via the tryptophan metabolic pathway.
Jiang et al. (2015)	96	China	2015	9.60	Article	Major depressive disorder is associated with gut microbiota alterations, including reductions in beneficial bacteria linked to symptom severity.
Erny et al. (2015)	95	Germany	2015	9.50	Article	The key regulatory role of microbial metabolites in neuroimmune cells
Zheng et al. (2016)	94	China	2016	10.44	Article	Gut microbial in depressed patients can causally induce depression-like behaviors through metabolic pathways.

### 3.7 Research frontiers: author keyword analysis

**Figure 7:**
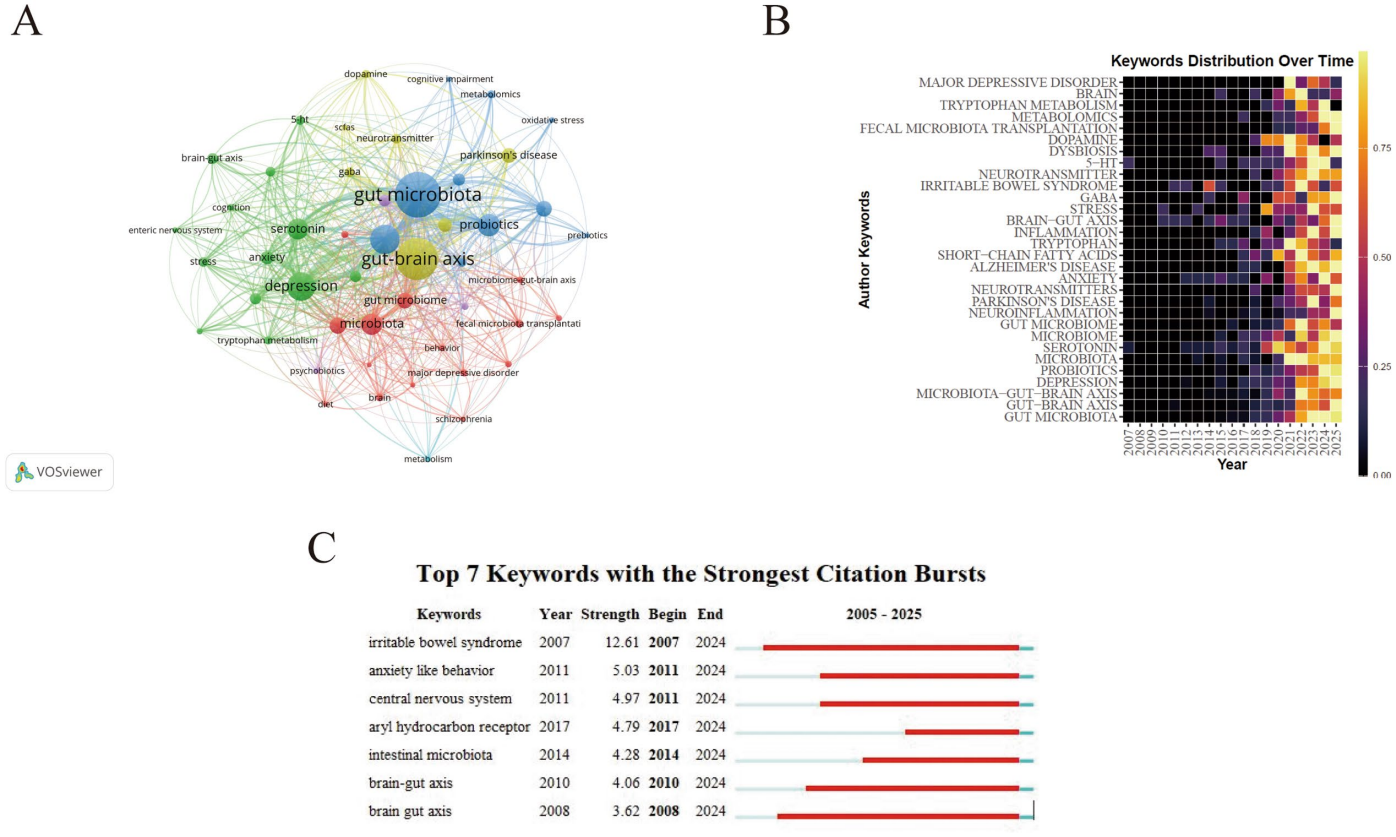
Keyword-based thematic structure and emerging topics in neurotransmitter-related gut–brain axis research. **(A)** Keyword co-occurrence network (VOSviewer). Each node represents an author keyword; node size is proportional to keyword occurrence frequency. Links represent keyword co-occurrence, with thicker links indicating stronger co-occurrence strength. **(B)** Author keywords distribution over time (heatmap). Rows represent author keywords and columns represent years; color intensity indicates the normalized frequency (0–1) of each keyword in a given year (higher intensity = higher relative frequency), illustrating temporal shifts in research attention. **(C)** Top 7 keywords with the strongest citation bursts. The “strength” value reflects burst intensity; red bars indicate the time intervals during which a keyword exhibits a citation burst, with burst begin and end years shown on the 2005–2025 timeline.

**Figure 8:**
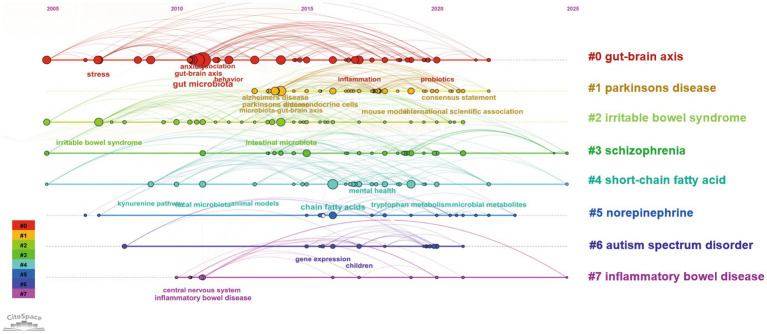
CiteSpace timeline visualization of co-citation clusters (2005–2025). Each horizontal line represents a thematic co-citation cluster (labels shown on the right). The *x*-axis indicates publication year. Nodes represent cited references within each cluster; node size is proportional to citation frequency in the dataset. Links indicate co-citation relationships, and the timeline layout shows how clusters emerge, persist, and evolve over time. Cluster colors correspond to the cluster identity as shown in the legend.

Keywords provide concise representations of research content and can help reveal the knowledge structure and thematic relationships within a field. As shown in Figure 7A keywords are visualized as nodes where node size corresponds to occurrence frequency. The 10 most frequent keywords were gut microbiota (*n* = 266) gut–brain axis (*n* = 220) depression (*n* = 165) microbiota (*n* = 154) serotonin (*n* = 121) microbiota–gut–brain axis (*n* = 113) anxiety (*n* = 112) probiotics (*n* = 107) short-chain fatty acids (*n* = 102) and inflammation (*n* = 100). Notably 16 of the top 20 high-frequency keywords also appeared among the top 30 overall indicating sustained attention to a stable core theme set. Temporal evidence from multiple bibliometric outputs collectively suggests a coherent frontier-shift pattern rather than isolated emerging terms. To avoid overgeneralization we explicitly distinguish between emerging themes and nascent signals. In this study emerging themes refer to keywords with relatively higher overall occurrence and consistent recent prominence in the heatmap/burst outputs whereas nascent signals refer to low-frequency terms that appear predominantly in the most recent time slices and may represent early-stage directions. Under this distinction topics such as neuroinflammation/metabolomics and intervention-related terms (e.g., fecal microbiota transplantation) can be interpreted as emerging themes while low-frequency terms such as ‘precision psychiatry’ and ‘vagus nerve stimulation’ are better treated as nascent signals that warrant monitoring in future updates. The temporal heatmap (Figure 7B) shows that keyword activity increased markedly after 2021 and remained high approaching 2025 reflecting intensified research attention in the recent period (noting that 2025 is an incomplete year due to the search cut-off). Burst detection (Figure 7C) further identifies keywords with rapidly increasing attention over specific time windows: irritable bowel syndrome exhibited the strongest burst intensity (12.61) with a long burst period from 2007 to 2024 indicating a persistent clinical anchor in the gut–brain axis literature. In contrast brain–gut axis although appearing early (2008) showed delayed prominence suggesting a gradual consolidation of terminology and research focus. Importantly integrating the heatmap trend (Figure 7B) burst signals (Figure 7C) and thematic evolution results (Figure 8) provides convergent support for a recent transition toward mechanistic–translational frontiers after 2020. Specifically the post-2020 period is characterized by increasing emphasis on neuroimmune mechanisms and multi-omics-enabled inference (e.g., neuroinflammation and metabolomics) alongside clinically oriented interventions and disease contexts (e.g., fecal microbiota transplantation neurodevelopment-related themes and neurodegenerative disorders such as Parkinson’s disease and Alzheimer’s disease). Emerging mechanistic directions such as dopamine-related themes and aryl hydrocarbon receptor–linked pathways also appear more prominently in the most recent time slices consistent with a shift from broad mechanistic exploration toward pathway-focused and translational studies. In addition microbiome modulation strategies (including probiotics/psychobiotics) and vagal signaling are increasingly discussed as intervention- and pathway-related directions; however terms such as “precision psychiatry” and “vagus nerve stimulation” have not yet become high-frequency author keywords in our dataset suggesting that they may represent nascent frontiers that are still gaining visibility.

### 3.8 Evolution of emerging research themes

Based on co-citation cluster analysis conducted in CiteSpace on literature from 2005 to 2025, this study identified seven major thematic clusters, with their temporal evolution illustrated in Figure 8. The earliest clusters emerged around 2005, including Cluster 0 (gut–brain axis), Cluster 2 (irritable bowel syndrome), Cluster 3 (schizophrenia), and Cluster 4 (short-chain fatty acids), indicating that early research had already begun to explore the gut–brain axis from multiple perspectives, encompassing gastrointestinal disorders, psychiatric conditions, and microbial metabolites. Within Cluster 0, keywords such as stress, gut microbiota, and inflammation reflect a progression from initial studies on stress responses to more in-depth investigations of neuroimmune mechanisms. Subsequent research phases saw the emergence of additional thematic clusters, including Cluster 5 (norepinephrine), Cluster 6 (autism spectrum disorder), and Cluster 7 (inflammatory bowel disease), indicating an expansion of research focus to specific neurotransmitters, neurodevelopmental disorders, and chronic intestinal inflammation. Notably, Cluster 1 (Parkinson’s disease), which appeared most recently, suggests that research on the association between Parkinson’s disease and the gut–brain axis began later but has gradually become an emerging focal point. Overall, over the past two decades, studies in this field have shown a clear evolutionary trend, moving from investigations of fundamental mechanisms toward disease-specific and neurotransmitter-targeted research. This evolution is consistent with the frontier synthesis in Section 3.7, where burst signals and temporal keyword intensification jointly indicate a recent shift toward neuroimmune mechanisms and clinically oriented translational themes.

## 4 Discussion

### 4.1 General condition

This bibliometric analysis maps neurotransmitter-related gut–brain axis research from 2005 to 2025 and reveals three broad phases: an exploratory stage (2005–2016) with relatively low output, a consolidation stage (2017–2019) with increased activity, and a rapid expansion stage (2020–2025) characterized by sharp growth and greater thematic diversification. Notably, because the search was conducted on 1 November 2025, publications indexed in 2025 represent an incomplete year; thus, annual counts for 2025 may be underestimated and any trend modeling should be interpreted primarily as descriptive rather than predictive. The marked post-2020 acceleration may reflect several converging drivers. Methodologically, wider access to high-throughput microbiome profiling and increasingly standardized bioinformatic workflows has enabled larger cohorts and more reproducible analyses, while the growing adoption of multi-omics integration (e.g., metagenomics and metabolomics) supports more mechanism-oriented studies linking microbial functions to neuroactive and immune pathways. Conceptually and translationally, expanding interest in psychiatric microbiome research and microbiome-targeted interventions has broadened the clinical relevance of gut–brain axis research. Importantly, this post-2020 acceleration is not only conceptual but is also reflected in our bibliometric outputs. As shown by the keyword heatmap/burst analysis and thematic evolution (Figures 7, 8), recent growth coincides with increased attention to neuroimmune mechanisms and multi-omics-enabled inference (e.g., neuroinflammation and metabolomics), alongside more disease- and intervention-oriented themes (e.g., fecal microbiota transplantation and neurodegenerative contexts). These shifts align with the maturation and wider accessibility of high-throughput sequencing and standardized microbiome bioinformatics workflows, and with increasing emphasis on mechanistic and translational work linking microbial functions to specific neural and immune pathways and potential therapeutic strategies.

Geographic and institutional analyses show that while research is globally distributed, outputs and collaborations are concentrated in a subset of countries and institutions. China contributes the largest publication volume (375 papers). The United States shows strong international collaboration connectivity (e.g., higher total link strength in co-authorship networks), indicating an important role in cross-country research exchange. China’s dominance in publication volume may reflect broader contextual drivers, including rapid expansion of biomedical research capacity and interdisciplinary programs, increased support for microbiome-related and brain/mental-health research, and wider access to high-throughput sequencing platforms and bioinformatics infrastructure that facilitate large-scale studies. In addition, large clinical populations and multi-center hospital networks may enable efficient cohort recruitment and translational projects that generate publishable outputs. Importantly, bibliometric indicators alone cannot establish causal links to specific funding or geopolitical mechanisms; therefore, these factors are discussed as plausible contributors rather than definitive explanations, and publication volume is interpreted alongside collaboration connectivity and citation-based indicators. Ireland’s citation impact relative to publication volume can be partly explained by the concentration of highly cited outputs from a small number of groups, particularly University College Cork (UCC). Institution-level results show that UCC accounts for 28 publications and 10,935 citations, indicating that a substantial fraction of Ireland’s citation impact is driven by this single institution. At the national level, Ireland shows high citation impact not only in total citations (14,449) but also in citations per publication (CPP = 412.83), indicating a disproportionately high citation yield relative to publication volume, whereas China is characterized by high output volume with a lower CPP (36.21). Moreover, five of the top 10 most-cited references in this field originate from Ireland, including highly cited integrative reviews and mechanistic studies associated with UCC-affiliated authors (e.g., Cryan, Dinan, and Clarke). Author-level analysis further indicates that John F. Cryan, Timothy G. Dinan, and Gerard Clarke rank among the most productive and highly cited contributors within the dataset. Keyword co-occurrence and burst analyses highlight sustained attention to core concepts such as gut microbiota, gut–brain axis, serotonin, depression, and short-chain fatty acids, indicating continued interest in molecular and behavioral mechanisms linking the microbiota to central nervous system function. Temporal trends suggest that research focus has gradually shifted from foundational mechanistic studies toward clinically relevant topics, including neurodevelopmental disorders, psychiatric conditions, and inflammatory bowel diseases. Co-citation cluster and thematic evolution analyses support this progression, showing early emphasis on stress, irritable bowel syndrome, schizophrenia, and microbial metabolites, followed by the emergence of clusters related to norepinephrine, autism spectrum disorder, and Parkinson’s disease. Collectively, these results depict a field evolving from mechanistic exploration to disease-specific and neurotransmitter-targeted research. Although our search strategy explicitly included acetylcholine- and histamine-related terms, these neurotransmitter systems did not appear among the most frequent author keywords nor as major clusters in the keyword co-occurrence and thematic evolution analyses. It should also be noted that terminology heterogeneity may reduce the visibility of these systems in keyword-based analyses. For example, cholinergic mechanisms are often discussed using broader or receptor-level terms (e.g., “cholinergic signaling,” “nicotinic/muscarinic receptors,” or “vagal cholinergic pathways”) rather than explicitly using “acetylcholine”; similarly, histamine-related studies may be indexed under “histaminergic” signaling or receptor-level terms (e.g., H1/H2 receptors). Therefore, the apparent underrepresentation may partly reflect differences in terminology and indexing practices in addition to true research imbalance. This pattern suggests that, compared with serotonin-, GABA-, and dopamine-centered themes, cholinergic and histaminergic mechanisms remain relatively underrepresented in the current gut–brain axis literature captured by our dataset. Several factors may contribute to this imbalance. Historically, research has focused on neurotransmitter pathways with well-established gut-derived precursor routes and clearer links to behavioral phenotypes (e.g., tryptophan–serotonin and GABA-related mechanisms), which may yield more readily testable hypotheses and standardized measurement approaches. In contrast, acetylcholine- and histamine-related signaling involves complex host–microbe–immune interactions and distributed receptor systems, making causal attribution and biomarker quantification more challenging. As multi-omics and targeted metabolomic profiling become more standardized, systematic investigation of these less-studied neurotransmitter systems may represent an important opportunity for future mechanistic and translational research. Analysis of highly cited journals and references illustrates the consolidation of a core knowledge base spanning neuroscience, psychiatry, microbiology, nutrition, and immunology. Journals such as Nutrients serve as prolific publication venues and highly cited sources within the dataset, while highly cited works from institutions in Ireland and China provide conceptual and mechanistic insights into the microbiota–neurotransmitter–brain interface. These studies offer widely used frameworks that guide subsequent research, particularly in linking molecular mechanisms to behavioral outcomes, thereby underpinning the ongoing evolution of the field.

### 4.2 Research hotspots: microbial regulation of neurotransmitters in the gut–brain axis

The gut microbiota establishes a stable symbiotic relationship with its host, producing a wide array of neuroactive compounds including neurotransmitters and their precursors during metabolic processes that play a pivotal role in gut–brain axis signaling (Deng et al., 2021). These microbial-derived molecules can reach the central nervous system via circulatory or neural pathways, modulating the concentrations of neurotransmitters and their precursors in the brain, thereby directly or indirectly influencing neural activity (Wong and Chiu, 2025). Neurotransmitters serve as chemical messengers for synaptic communication between neurons, regulating critical functions such as motor control, emotional processing, and cognitive performance, and exerting either excitatory or inhibitory effects on target neurons (Shi et al., 2025). In gut–brain interactions, certain neurotransmitters mediate the interplay between microbes and the host, with their synthesis being modulated by microbial metabolism of precursor substances. Normal brain function relies on complex neurotransmitter-mediated signaling between neurons and glial cells, and dysregulation of major neurotransmitter systems including dopaminergic, cholinergic, serotonergic, glutamatergic, and GABAergic pathways has been closely associated with the pathogenesis of various central nervous system disorders (Teleanu et al., 2022; Franco et al., 2021; Satarker et al., 2022). Increasing evidence demonstrates that the gut microbiota can influence brain function by modulating neurotransmitter balance, highlighting its potential as a regulatory target and suggesting novel strategies for intervention in CNS diseases (Chen et al., 2021; Strandwitz, 2018). The regulation of host neurotransmitters and their associated pathways by the microbiota represents a central mechanism mediating bidirectional communication within the gut–brain axis. Numerous studies have confirmed that the gut microbiota can synthesize multiple key neurotransmitters, a discovery that has even contributed to the emergence of microbial endocrinology as an independent discipline (Cotton et al., 2023). High-frequency keywords such as serotonin, norepinephrine (cluster #5), and short-chain fatty acids (cluster #4) indicate that microbial modulation of neurochemical signaling remains a core focus of the field. Accordingly, to provide a comprehensive overview, this study summarizes key data on neurotransmitters produced or directly influenced by the gut microbiota (Table 5), demonstrating that these microbial-derived molecules play a central role in shaping host neurochemical networks.

**Table 5:** Bacterial production of neurotransmitters.

Neurotransmitter	Bacterial genus
5-HT	*Lepidium* ssp. (Hong et al., 2023)
	*Lactobacillus* ssp. (Huang et al., 2023)
	*Escherichia* ssp. (Nzakizwanayo et al., 2015)
Dopamine	*Bacillus* ssp. (Rehman et al., 2022)
	*Escherichia* ssp. (Scott et al., 2024)
	*Klebsiella* ssp. (Li et al., 2024)
	*Proteus* ssp. (Zhao et al., 2025)
Norepinephrine	*Bacillus* ssp. (Rehman et al., 2022)
	*Escherichia* ssp. (Sule et al., 2017)
	*Proteus* ssp. (Tsavkelova et al., 2000)
	*Serratia* ssp. (Tsavkelova et al., 2000)
GABA	*Bacteroides* ssp. (Strandwitz et al., 2019)
	*Bifidobacterium* spp. (Altaib et al., 2022)
	*Akkermansia* spp (Li et al., 2022)
	*Clostridium* spp (Hardman and Stadtman, 1960)
	*Adlercreutzia* spp. (Weng et al., 2023)
	*Alistipes* spp. (Bajaj et al., 2021)
	*Lactobacillus* spp. (Bravo et al., 2011)
	Parabacteroides ssp. (Strandwitz et al., 2019)
	*Eubacterium* ssp. (Strandwitz et al., 2019)
Glutamate	*Lactobacillus* ssp. (Teixeira et al., 2014)
	*Bacteroides* ssp. (Liu et al., 2017)
	*Campylobacter* ssp. (Van Der Stel et al., 2015)
Acetylcholine	*Lactobacillus* spp. (Stanaszek et al., 1977)

### 4.3 The critical role of the neuroimmune axis in gut–brain communication

**Figure 9:**
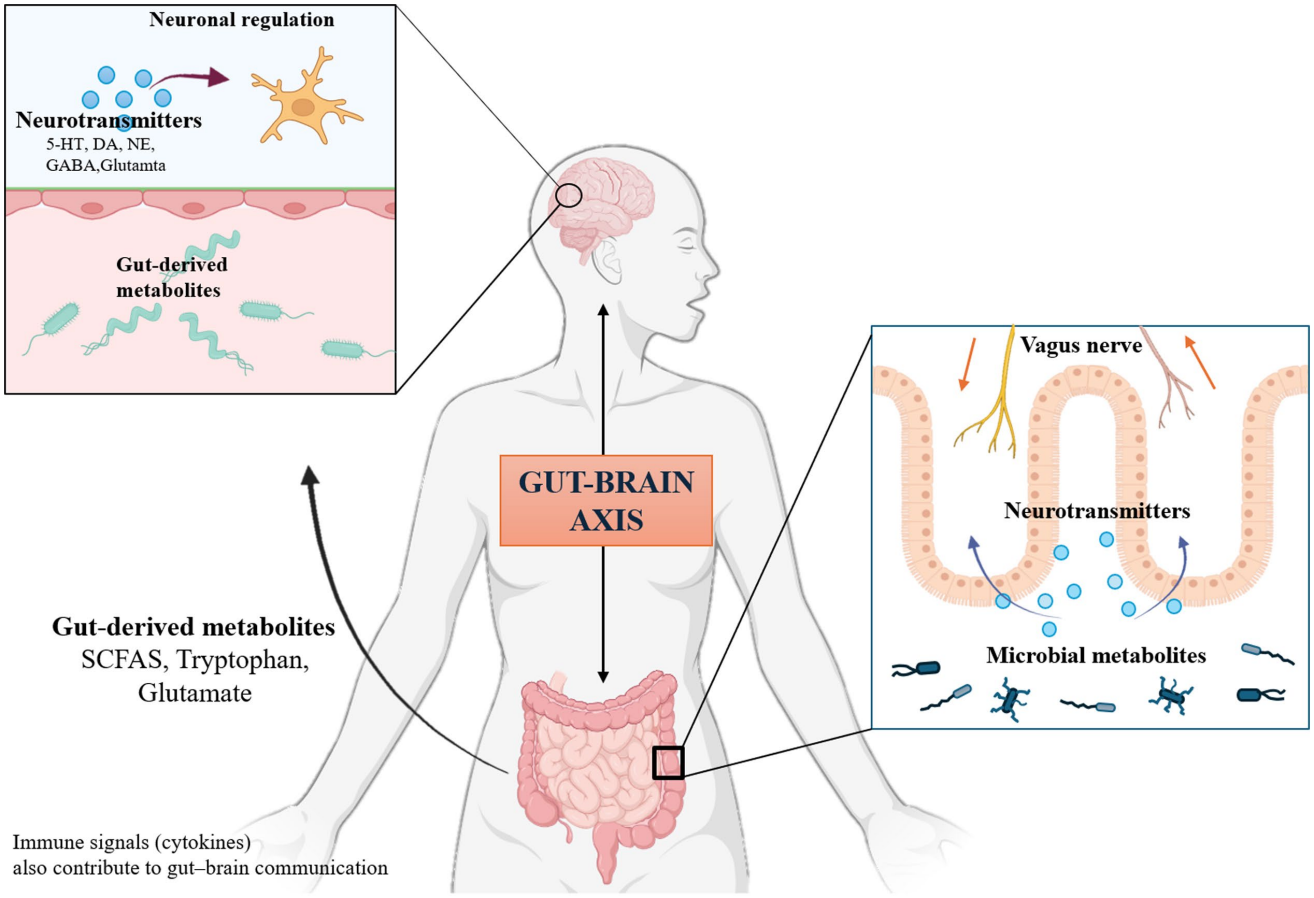
Mechanistic pathways linking gut microbiota-derived metabolites and neurotransmitters to central nervous system regulation.

Inflammation has consistently emerged as a core theme in gut–brain axis research, reflecting the foundational role of neuroimmune mechanisms. As illustrated in Figure 9, the gut–brain axis, a bidirectional network linking the gut microbiota and the central nervous system, is tightly regulated by neuroimmune processes, with microglia serving as key mediators (Nayak et al., 2014; Prinz et al., 2019). Evidence indicates that gut dysbiosis and increased intestinal permeability often precede neurodegenerative pathology, facilitating microbial metabolite entry into circulation, enhancing blood–brain barrier permeability, and priming central neuroinflammation (Derkinderen et al., 2025). The gut, as the largest immune organ and home to the enteric nervous system, relies on enteric glial cells to maintain epithelial integrity, coordinate enteric reflexes, and modulate local immunity. Reactive enteric glial activation represents an early link between intestinal inflammation and central pathology (Agirman et al., 2021; Jacobson et al., 2021). Gut-derived signals converge on microglia to induce neuropathological changes through diverse mechanisms (Wallrapp and Chiu, 2024). In neurodevelopment, early-life gut dysbiosis can disrupt the balance of neuroactive compounds, including short-chain fatty acids and serotonin, triggering low-grade neuroimmune inflammation that affects microglia-mediated synaptic pruning and neural circuit maturation, ultimately leading to behavioral abnormalities (Hughes et al., 2023; Fan et al., 2023). In neurodegeneration, Parkinson’s disease pathology supports the gut-origin hypothesis, wherein dysbiosis or gut inflammation promotes *α*-synuclein misfolding and aggregation in the enteric nervous system, which can propagate via the vagus nerve to the substantia nigra, directly damaging dopaminergic neurons and activating microglia to exacerbate neuroinflammation and oxidative stress (Saleh et al., 2022; Zhang et al., 2023; Salim et al., 2023; Claudino Dos Santos et al., 2023). Together, autism and Parkinson’s disease exemplify how the gut–brain axis and neuroimmune signaling contribute to both neurodevelopmental and neurodegenerative disorders, underscoring the axis’s central role in CNS disease and highlighting key directions for future research.

### 4.4 The gut–brain axis research ideal model

Cluster 7 in the thematic evolution trajectory highlights inflammatory bowel disease (IBD) as an ideal human model for investigating gut–brain axis mechanisms. Clinically, IBD patients frequently exhibit neuropsychiatric comorbidities, including anxiety, depression, and cognitive fatigue, at rates significantly higher than the general population (Bisgaard et al., 2022). These manifestations cannot be solely attributed to the psychological burden of chronic illness, indicating a distinct biological underpinning. Consequently, IBD provides a natural model to explore gut-driven brain dysfunction and offers a unique framework for dissecting pathophysiological mechanisms originating in the gut (Gracie et al., 2019). Immune-inflammatory pathways are central to this process. Proinflammatory cytokines produced locally in the IBD-affected intestine can breach a compromised gut barrier, enter systemic circulation, and disrupt blood–brain barrier integrity, thereby activating microglia and precipitating neuroinflammation alongside neurotransmitter system dysregulation (Fakhfouri et al., 2024). Neuroendocrine dysregulation also contributes, as chronic intestinal inflammation can perturb hypothalamic–pituitary–adrenal axis function, generating a vicious cycle that exacerbates both intestinal pathology and systemic stress responses (Bonaz and Bernstein, 2013). Microbiota-mediated mechanisms further modulate these processes. IBD-associated dysbiosis alters the production of microbial metabolites, such as short-chain fatty acids, which maintain gut barrier integrity and can traverse the blood–brain barrier to influence microglial activation and neuronal signaling (Petracco et al., 2025). Additionally, gut-derived neurotransmitters, particularly serotonin, may act as critical mediators linking intestinal dysfunction to central neural disturbances. These insights underscore the translational potential of IBD as a model for gut-brain axis research. Future studies should prioritize evaluating immunomodulatory therapies for neuropsychiatric comorbidities and investigating microbiome-based interventions. Collectively, IBD offers a distinctive and integrative platform connecting intestinal immune dysregulation to brain dysfunction, thereby illuminating the complex mechanisms underpinning gut–brain communication.

### 4.5 Limitations

Although this study provides a comprehensive bibliometric overview of neurotransmitter-related gut–brain axis research, several limitations should be acknowledged. First, database coverage and language restrictions may introduce selection bias because the dataset was limited to WoSCC, Scopus, and PubMed and to English-language articles/reviews; thus, relevant studies indexed in other databases (e.g., Embase) or published in other languages may have been missed, potentially affecting global representativeness and regional comparisons. Second, we focused on peer-reviewed articles and reviews and excluded gray literature (e.g., conference proceedings, book chapters, and preprints), which may contain early signals of emerging topics and could lead to delayed detection of nascent trends. Third, bibliometric outputs are sensitive to analytical settings and tool-specific algorithms (e.g., clustering and labeling strategies in CiteSpace/VOSviewer), and therefore some differences in cluster boundaries or keyword prominence may occur; future work could further validate robustness by systematically varying thresholds and comparing outputs across tools. Fourth, citation-based indicators are influenced by publication age, document type (reviews vs. original research), journal visibility, and self-citation behavior. Self-citations were not systematically excluded due to inconsistent citing–cited linkage and author disambiguation across merged databases; therefore, citation metrics should be interpreted cautiously. To mitigate age bias, we additionally reported normalized metrics (e.g., citations per year for highly cited references and citations per publication for country-level comparisons), but recently published high-impact studies may not yet have accumulated sufficient citations to be highlighted. Although self-citations were not systematically removed, their impact is expected to be more pronounced in fine-grained author-level analyses than in aggregated country/institution-level summaries. Nevertheless, self-citation could still influence close rankings among entities with similar citation totals; therefore, national and institutional comparisons should be interpreted as approximate indicators of influence rather than definitive performance metrics. Fifth, because the search cut-off was 1 November 2025, 2025 represents an incomplete year due to indexing lag, which may underestimate annual output and affect trend visualization. Finally, the growth model was used as a descriptive summary of historical patterns rather than a predictive tool and should not be interpreted as deterministic forecasting. Future work could expand database coverage, incorporate multilingual sources, and apply more systematic sensitivity analyses to further strengthen robustness and generalizability.

## 5 Conclusion

This study provides the first comprehensive bibliometric analysis of neurotransmitter-related gut-brain axis research, mapping its developmental trajectory, key research areas, and emerging trends. The findings provide objective data support and a macroscopic perspective to help scholars grasp the overall knowledge structure and track the evolution of core topics. Based on the findings, future research should focus on integrating multidisciplinary approaches, especially targeting neurotransmitter pathways and considering individual microbial profiles. Additionally, leveraging advanced techniques like metagenomics, metabolomics, and host genomics will help translate mechanistic insights into novel therapeutic strategies for neuropsychiatric disorders. From a translational perspective, there is a pressing need to develop precision interventions targeting neurotransmitter pathways while accounting for individual microbial profiles, thereby advancing the field from mechanistic understanding toward novel therapeutic strategies for neuropsychiatric disorders.

## Data Availability

The original contributions presented in the study are included in the article/Supplementary material, further inquiries can be directed to the corresponding author.
