# Supplementary material for: Neurotransmitter-mediated gut-brain axis: a bibliometric analysis of research trends and knowledge structure
Source: Front Microbiol. 2026 Apr 13;17:1771169. doi: 10.3389/fmicb.2026.1771169 (PMC13111198; doi:10.3389/fmicb.2026.1771169)
Supplement: Supplementary file 1 [file Table_1.docx]

| Scopus | #1 TITLE-ABS-KEY=("gut-brain axis" OR "microbiota-gut-brain axis" OR "microbiome-gut-brain axis" OR "brain-gut axis" OR "gut-brain communication") |
| --- | --- |
|  | #2 TITLE-ABS-KEY= ("neurotransmitter*" OR "serotonin" OR "5-HT" OR "dopamine" OR "GABA" OR "glutamate" OR "norepinephrine" OR "noradrenaline" OR "acetylcholine" OR "histamine"OR"neurotransmitter precursor" OR "tryptophan" OR "tyrosine" OR "glutamic acid" OR OR "choline" OR "dopa precursor") |
|  | #3 Document Type=Article or Review |
|  | #4 Language=English |
|  | #5 PY=（2005-2025） |
|  | #6 #1 AND #2 AND #3 AND #4 AND #5 |
| Web of Science Core Collection | #1 TITLE-ABS-KEY=("gut-brain axis" OR "microbiota-gut-brain axis" OR "microbiome-gut-brain axis" OR "brain-gut axis" OR "gut-brain communication") |
|  | #2 TITLE-ABS-KEY= ("neurotransmitter*" OR "serotonin" OR "5-HT" OR "dopamine" OR "GABA" OR "glutamate" OR "norepinephrine" OR "noradrenaline" OR "acetylcholine" OR "histamine"OR"neurotransmitter precursor" OR "tryptophan" OR "tyrosine" OR "glutamic acid" OR OR "choline" OR "dopa precursor") |
|  | #3 Document Type=Article or Review |
|  | #4 Language=English |
|  | #5 PY=（2005-2025） |
|  | #6 #1 AND #2 AND #3 AND #4 AND #5 |
| Pubmed | #1 ("gut-brain axis" OR "microbiota-gut-brain axis" OR "microbiome-gut-brain axis" OR "brain-gut axis" OR "gut-brain communication") |
|  | #2 ("neurotransmitter*" OR "serotonin" OR "5-HT" OR "dopamine" OR "GABA" OR "glutamate" OR "norepinephrine" OR "noradrenaline" OR "acetylcholine" OR "histamine"OR"neurotransmitter precursor" OR "tryptophan" OR "tyrosine" OR "glutamic acid"OR "choline" OR "dopa precursor") |
|  | #3 Document Type=Article or Review |
|  | #4 Language=English |
|  | #5 PY=（2005-2025） |
|  | #6 #1 AND #2 AND #3 AND #4 AND #5 |
